# Supplementary material for: The interactome of intact mitochondria by cross-linking mass spectrometry provides evidence for coexisting respiratory supercomplexes
Source: Mol Cell Proteomics. 2017 Dec 8;17(2):216–32. doi: 10.1074/mcp.RA117.000470 (PMC5795388; doi:10.1074/mcp.RA117.000470)
Supplement: Supplemental Data [file supp_RA117.000470_133922_0_supp_23978_fzffwf.zip › spectra_annotation/mito_DR_spectra_annotation/434-1-10-1-5-1.pdf]

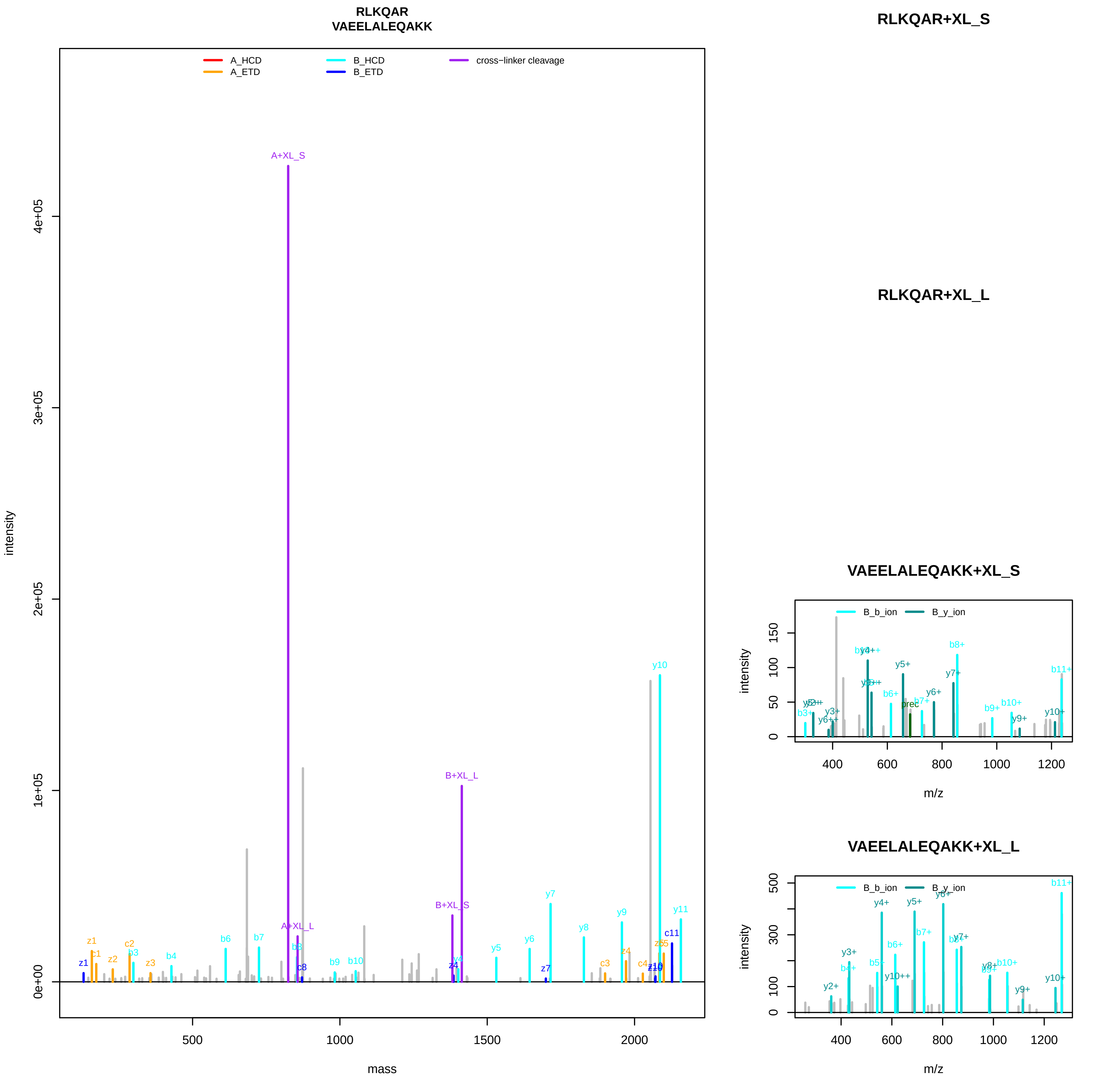

RLKQAR
VAEELALEQAKK
A_HCD
A_ETD
B_HCD
B_ETD
cross−linker cleavage
A+XL_S
A+XL_L
B+XL_L
B+XL_S
intensity
mass
0e+00
1e+05
2e+05
3e+05
4e+05
500
1000
1500
2000
y10
y11
y9
y8
y7
y6
y5
c11
z7
z4
c8
b10
b9
b8
b7
b6
b4
b3
z1
z2
z3
c1
c2
c3
c4
z4
RLKQAR+XL_S
RLKQAR+XL_L
VAEELALEQAKK+XL_S
VAEELALEQAKK+XL_L
B_b_ion
B_y_ion
m/z
prec
